# The involvement of the vasa vasorum in the development of vasculitis in animal model of Kawasaki disease

**DOI:** 10.1186/1546-0096-12-12

**Published:** 2014-03-30

**Authors:** Akiko Hamaoka-Okamoto, Chinatsu Suzuki, Tomoyo Yahata, Kazuyuki Ikeda, Noriko Nagi-Miura, Naohito Ohno, Yoshinori Arai, Hideo Tanaka, Tetsuro Takamatsu, Kenji Hamaoka

**Affiliations:** 1 Department of Pediatric Cardiology and Nephrology, Kyoto Prefectural, University of Medicine Graduate School of Medical Science, Kamigyo-ku, Kyoto 602-8566, Japan; 2 Laboratory for Immunopharmacology of Microbial Products, School of Pharmacy, Tokyo University of Pharmacy and Life Sciences, Hachioji, Tokyo 192-0392, Japan; 3 Nihon University School of Dentistry, Chiyoda-ku, Tokyo 101-8310, Japan; 4 Department of Pathology and Cell Regulation, Kyoto Prefectural University of Medicine Graduate School of Medical Science, Kamigyo-ku, Kyoto 602-8566, Japan

**Keywords:** Kawasaki disease, A murine model, Vasculitis, Adventitia, Vasa vasorum

## Abstract

**Background:**

Kawasaki Disease (KD) involves a diffuse and systemic vasculitis of unknown etiology that mainly affects infants and children. Although a considerable number of analyses of the clinical, histopathological and molecular biological details underlying the mechanism responsible for the development of coronary arterial lesions, it is still poorly understood.

The purpose of this study was to analyze the state of angiogenesis, vasculogenesis and the distribution of blood vessels using an animal model of KD like vasculitis. We investigated the involvement of the vasa vasorum from the adventitia in the vascular involvement and the development of the disease state by performing sequential histopathology, scanning electron microscopy (SEM) and micro computed tomography (CT) studies using a murine model of vasculitis induced by the *Candida albicans* water-soluble fraction (CAWS).

**Methods:**

To prepare the animal model of KD like vasculitis, CAWS was intraperitoneally injected into C57BL/6N mice for five consecutive days as reported by *Ohno et al*. We observed the changes of the vasa vasorum at the aorta and the orifices of the coronary arteries by SEM and micro CT, and also compared the neovascularization at the media and adventitia of the aorta by an immunohistochemical analysis.

**Results:**

As previously reported, obvious inflammation was detected two weeks after the injection of CAWS, and also intimal thickening was observed three weeks after the injection. We found that the vasa vasorum in the adventitia of the aorta was increased in the model mice. The vasa vasorum started increasing one week after the injection of CAWS, before any obvious vasculitis was microscopically detected.

**Conclusion:**

The present results indicate that the vasculitis in Kawasaki disease starts as a disorder of the vasa vasorum.

## Background

Kawasaki disease (KD) involves diffuse, systemic vasculitis of unknown etiology and pathogenesis, and predominantly affects infants and children [1-3]. The incidence of KD has steadily increased since it was first reported, and more than 12,000 people are diagnosed with KD each year in Japan. The well-known sequelae of KD include coronary aneurysms, which occur in approximately 5% of the KD patients [4]. With time, an aneurysm may cause stenotic lesions or ischemic heart disease, even in children [5-7].

Intravascular immunoglobulin (IVIG) therapy can greatly decrease the chances of complications of coronary arterial lesions (CALs), though 10–15% of the patients are refractory to IVIG. In these cases, the incidence of CALs tends to be much higher.

Since KD was first described by Kawasaki in 1967, many clinical, histopathological and or molecular biological studies have been performed to investigate the pathophysiology of the vascular involvement of KD; however, the underlying mechanism remains unclear [2,3,8,9]. Coronary arteritis in KD is considered to begin with edematous changes in the media, developing into inflammatory changes in the intima and adventitia, which finally evolve into the panvasculitis observed during autopsies [8].

In contrast, recent studies using various animal models of KD like vasculitis have indicated that the inflammatory changes in the adventitia occur prior to the changes in the intima [10-12]. In 1975, Onouchi et al. suggested that the vasa vasorum, which supplies nutrition to the walls of blood vessels, might contribute to the progression of vasculitis in KD, beginning with severe tissue destruction at the vasa vasorum in the adventitia [9]. Although there have not been detailed pathological studies on the significance of the vasa vasorum in KD, recent studies using the advanced imaging analyses have shown that the abnormal proliferation of the vasa vasorum was associated with plaque formation and the destabilization of lesions during the development of arterial sclerosis. In chronic inflammatory diseases such as arterial sclerosis, the abnormal proliferation of the vasa vasorum may provide an infiltrative route for inflammatory cells from the adventitia, and this accumulation of inflammatory cells promotes the inflammation [13-15]. Thus, we hypothesized that the vasa vasorum might serve as the initiator of vasculitis in KD.

Various animal models have been developed to investigate KD vasculitis. In 1979, Murata and Naoe et al. first reported that the *Candida albicans* derived substance (CADS) isolated from the feces of patients with KD causes coronary arteritis as seen in KD [16]. Subsequent studies revealed that the water-soluble extracellular polysaccharide fraction (CAWS) obtained from the culture supernatant of *Candida albicans* resulted in a stronger but similar vasculitis that occurred more frequently than that following exposure to CADS. Therefore, CAWS-induced vasculitis has become widely adopted as a universal animal model for KD like vasculitis [17-25].

The purpose of this study was to delineate the involvement of the vasa vasorum in the development of KD vasculitis by examining its proliferation and distribution using histopathological or micro-computed tomography (CT) and scanning electron microscope (SEM) studies. KD is associated with a low mortality rate, and due to the low availability of autopsied hearts, we used the CAWS animal model for this purpose.

## Methods

### Animals

All experimental procedures were approved by the Committee for Animal Research, Kyoto Prefectural University of Medicine. A total of 24 four-week-old male C57BL/6N mice were obtained from SHIMIZU Laboratory Supplies Co., Ltd. (Kyoto, Japan) and were randomly divided into six experimental groups: control and model mice evaluated at one week, two weeks and three weeks after the administration of CAWS.

CAWS was kind gift from Tokyo University of Pharmacy and Life Science and 0.2 mL (20 mg/mL) was administered intraperitoneally to the model mice daily for five consecutive days starting at the age of five weeks.

### Histopathology

After heparin was administered, anesthesia was induced with a fatal dose of pentobarbital sodium. The chest was then opened, and the abdominal aorta was cannulated and infused with saline until the venous effluent was free of blood. Sections of the heart at the level of the aortic valves were obtained.

Tissue specimens were fixed in 20% formalin, embedded in paraffin and sectioned at 4 μm-thick slices. First, hematoxylin and eosin (HE)- and elastic van Gieson (EVG)-stained sections were examined by light microscopy.

Next, immunohistochemical studies for CD3 and MPO were performed to detect the type of inflammatory cells. Some sections were deparaffinized in xylene and ethanol rinses. Only sections for CD3 were activated by boiling for 5 min in a citrate buffer (0.01 M) at pH 9.0 in a microwave. No pretreatment was needed for MPO. After blocking of non- specific binding, the sections were incubated at 4 °C overnight in a humidified slide chamber with polyclonal antibodies (Dako Cytomation) for CD3 (dilution 1:400) and MPO (dilution 1:600). After incubation with the corresponding secondary antibody for 30 minutes, the antibody binding was revealed using H_2_O_2_ and diaminobenzidine. Counterstaining was performed with hematoxylin, and sections were examined by light microscopy.

Immunofluorescent studies were also performed using Biotin-conjugated Isolectin IB_4_ (Molecular Probes, Inc.). Some sections were deparaffinized in xylene and ethanol rinses, and activated by boiling for 5 min in a citrate buffer (0.01 M) at pH 6.0 in a microwave. The sections were incubated at 4°C overnight in a humidified slide chamber with lectin diluted at 1:200. The stained specimens were observed using a confocal laser scanning microscope (LSM510 ver. 4.0; Carl Zeiss CO., Ltd., Oberkochen, Germany).

### SEM

To examine the types of vessels, we investigated the adventitia of the micro- vessels using SEM [26]. After administering lethal anesthesia with pentobarbital sodium, a blue synthetic resin (Mercox®, DIC, Tokyo) was injected through a 22 Ga plastic needle inserted into the left ventricle. The sample was maintained at room temperature until the liquid resin was completely polymerized. The heart, aorta and perivascular soft tissues were then harvested *en bloc*. The soft tissues were dissolved by incubation in 20% potassium hydroxide solution at 50°C for three days. To completely dissolve the fat tissues, the samples were incubated with proteinase K in buffer ATL for one day. Then, the samples were washed in 0.5% Nonidet P-40. The microvascular casts were mounted on stubs, coated with osmium and examined using SEM (JSM-6320 F; JEOL, Tokyo).

### Micro CT

After lethal anesthesia with pentobarbital sodium was induced, the thorax was opened. The abdominal aorta was cannulated and infused with heparinized saline until the venous effluent was free of blood. Next, the OMNIPAQUE 240 contrast agent was infiltrated from the abdominal aorta at a steady rate.

The aorta and coronary arteries were scanned *en bloc* by a micro CT system (Rigaku, Tokyo, Japan) [27-29]. The resulting three-dimensional images were displayed using the i-view software program (Morita, Kyoto, Japan). The number of pixels used to reconstruct the images was 500 × 500 × 500, and the time required for filming and reconstruction was two minutes. It was also possible to examine a three-dimensional tomographic image simultaneously. The pixel size was 10 μm, and the filming span was 5 mm × 5 mm.

## Results

### Histopathology

**Figure 1:**
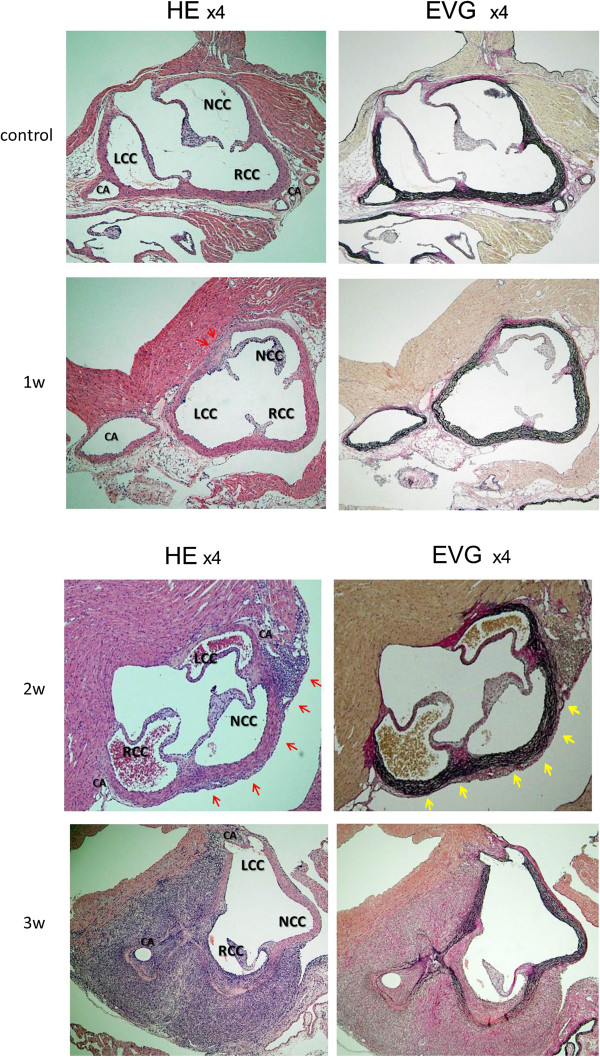
**The four developmental stages in the murine model of Kawasaki disease like vasculitis based on the histopathological analysis.** In hematoxylin and eosin (HE)-stained sections, there were some inflammatory cells visible in the adventitia one week after the CAWS injection (red arrows). Obvious infiltration of inflammatory cells was detected more than two weeks after the injections of the *Candida albicans* water-soluble fraction (CAWS) (red arrows). Three weeks after the injection, marked adventitial thickening was detected.In elastica van Gieson (EVG)-stained sections, the elastic fibers started to be destroyed more than two weeks after the injections of CAWS (yellow arrows). RCC: Right Coronary Cusp, LCC: Left Coronary Cusp, NCC: Non Coronary Cusp, CA: Coronary Artery.

One week after the CAWS injection, there were some inflammatory cells visible in the adventitia. Two weeks after the injection, there was clear infiltration of inflammatory cells and destruction of the elastic fibers. Marked adventitial thickening was detected at time points three or more weeks after the injection (Figure 1) [25].

**Figure 2:**
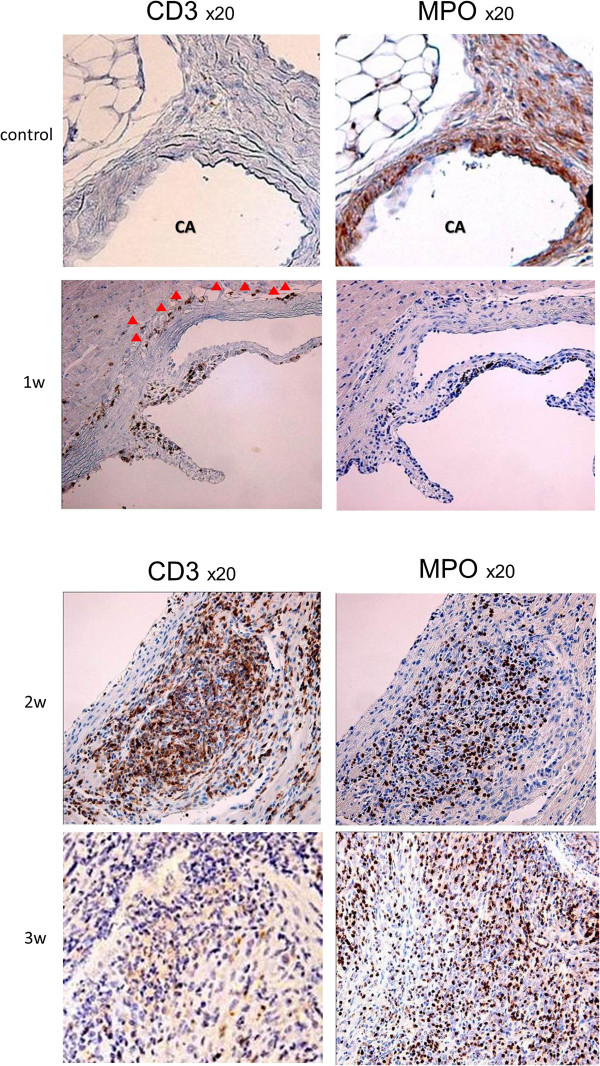
**The results of the sections stained with antibodies for CD3 and MPO.** In only sections with antibody for CD3, there were some CD3 positive cells visible at the same position inflammatory cells were detected one week after the *Candida albicans* water-soluble fraction (CAWS) injection (red triangles). More CD3 positive cells and some MPO positive cells were detected around coronary arteries more than two weeks after the injections of the injection. Three weeks after the injection, more MPO positive cells were detected.

Next, immunohistochemical studies were performed to observe the type of inflammatory cells using antibodies for CD3 and MPO. One week after the CAS injection, there were some CD3 positive cells only around aorta, at the same position inflammatory cells were detected by HE stain. Two weeks after the injection, more CD3 positive cells and some MPO positive cells were detected even around coronary arteries. More MPO positive cells were observed at time points three or more weeks after the injection (Figure 2). These results by HE stain and immunostain revealed the inflammatory cells consisted on monocytes, lymphocytes and neutrophils.

**Figure 3:**
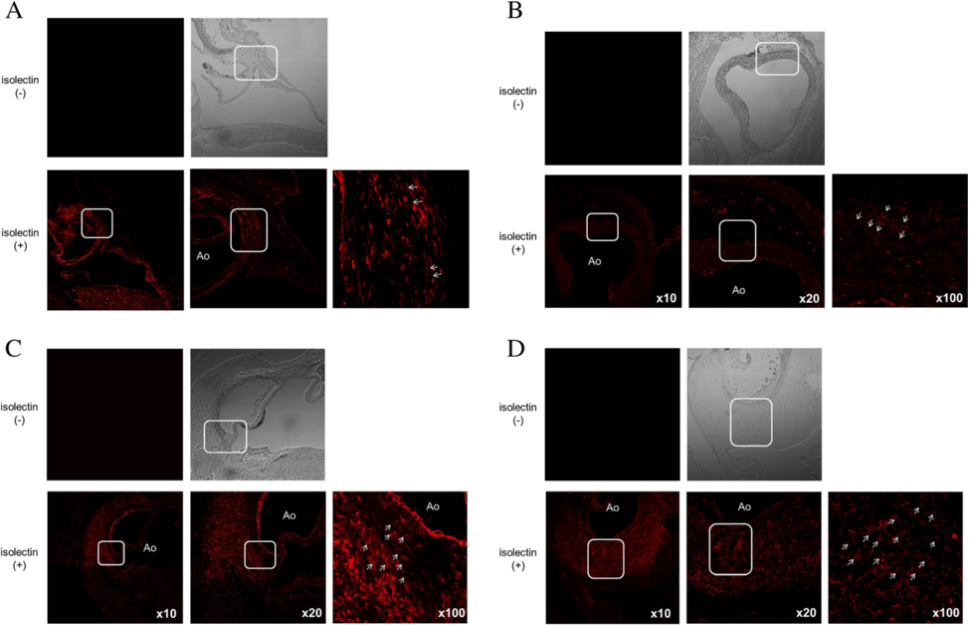
**The results of the sections stained with Isolectin. A**: A representative control mouse. Only a few Isolectin-positive endothelial cells were detected at the adventitia. **B**: A model mouse one week after the injection of *Candida albicans* water-soluble fraction (CAWS). Compared to the control, there were more Isolectin-positive endothelial cells detected at the adventitia. **C**: A model mouse two weeks after the injection of CAWS. The Isolectin-positive cells had infiltrated inward. **D**: A model mouse three weeks after the injection of CAWS. The Isolectin-positive cells infiltrated all layers.

Immunofluorescent studies were also performed to observe the appearance of the inflammatory cells at the adventitia and their infiltration inward, and we found small vessels that were not detected in control mice. Figure 3 shows the sections stained with Isolectin. Compared with the controls, some lectin- positive endothelial cells were visible only at the adventitia of the aorta one week after the injection of CAWS. Similar to the inflammatory cells, the lectin-positive cells had infiltrated inward two weeks after the injection, and by the following week, these cells filled all layers.

It has been demonstrated that the vasa vasorum provides a considerable amount of blood flow to the adventitia and one- third of the outer layer of the media [30-32]. Therefore, the blood vessels in the adventitia are part of the vasa vasorum or its branches.

**Figure 4:**
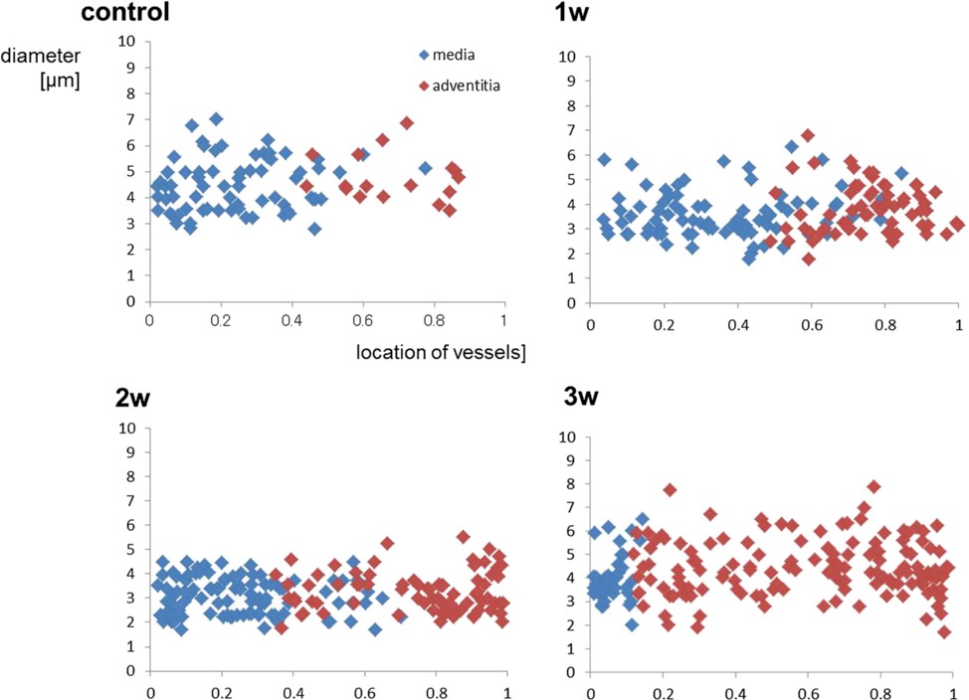
**The size and distribution of small vessels, including the vasa vasorum.** The X-axis shows the location of each vessel. “1” indicates external to the vessel, and “0” indicates the center of the lumen of that vessel.The Y-axis shows the size of vessels.The vessels proliferated at the adventitia along with the development of the inflammation.

We also examined the size and distribution of the vasa vasorum. As shown in Figure 4, the vasa vasorum proliferated as the inflammation developed.

### SEM

**Figure 5:**
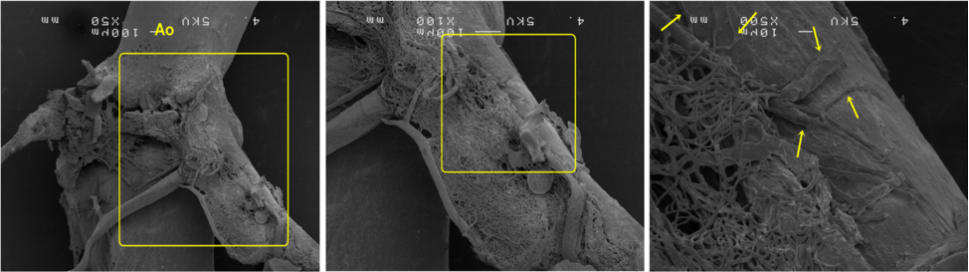
**An example of the small vessels around the aorta as examined by a scanning electron microscope (SEM).** These vessels surrounded the aorta and coronary arteries with a net-like appearance, and flowed into the host vessels (yellow arrows).

There were many vessels surrounding the aorta and coronary arteries in a net-like appearance. (Figure 5). These were considered to be part of the vasa vasorum, because they were arising from or invading the host vessels, as reported previously [28,33-35].

**Figure 6:**
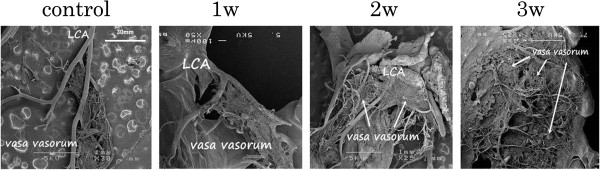
**The four developmental stages of vasculitis in the murine model of Kawasaki disease as examined using a scanning electron microscope (SEM).**The proliferation of the vasa vasorum was accompanied by an increase in the inflammation.

In the group with inflammation, the number of vasa vasorum increased. Furthermore, these networks of vasa vasorum proliferated as the infiltration of the inflammation increased (Figure 6).

### Micro CT

**Figure 7:**
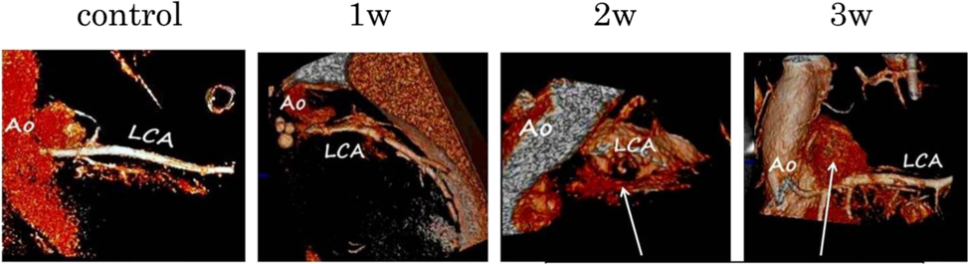
**The four developmental stages of vasculitis in the murine model of Kawasaki disease as examined by micro computed tomography (CT).**The aorta and the coronary arteries were detected. Beginning approximately two weeks after the injection of *Candida albicans* water-soluble fraction (CAWS), the bifurcation of the coronary arteries became unclear because of the leakage of the contrast agent.

On scanning the aorta and coronary arteries, the left coronary artery was found to clearly bifurcate from the aorta in the control group and in the animal model one week after the injection of CAWS. However, at more than two weeks after the injection, the bifurcation of the left coronary artery was no longer detectable due to the leakage of contrast agent (Figure 7). This suggested that the blood vessel wall was fragile due to the development of inflammation.

## Discussion

Kawasaki disease (KD) is characterized by systemic vasculitis with tissue edema at the initial phase of the disease. Although the inflammation is initially localized to the capillaries and microvessels, it eventually expands to medium-sized muscular arteries and veins. Our study found that the inflammation originated in the adventitia. The inflammation enhances the expression of growth factors and the resulting edema, leading to hypoxia due to the proliferation of the vasa vasorum (Figures 1, 2, 3). The proliferation of the vasa vasorum may act as a conduit for the entry of various growth factors [35], cytokines and blood cells. The spread of the proliferated vasa vasorum to the media thus broadens the inflammation.

In general, the inflammatory cells infiltrate from the adventitia toward the external elastic lamina around the Valsalva sinus and aortic valves by approximately one week after the administration of CAWS. During the second week, significant inflammatory changes develop from the adventitia and media toward the intima, as shown in Figures 1, 2 and 3, especially around the right and left coronary sinuses. The inflammatory cells, predominantly consisting of polymorphonuclear neutrophil leukocytes, are observed along the elastic lamina. More than three weeks later, the inflammation expands circumferentially to include the non-coronary sinus, and the beginning of an abscess can be observed. Fibrinoid necrosis is detected in the internal elastic lamina, and the intima and the adventitia markedly thicken. Therefore, the basic structure of blood vessels is destroyed, and they become fragile.

In the very early phase of inflammation, although the inflammatory cells accumulate at the adventitia, the detachment of endothelial cells or a thrombus indicative of the damaged intima are not detected. This suggests that the vasculitis is initiated from the side of the adventitia [36].

As the inflammatory cells infiltrate through the vasa vasorum, the neogenesis of more vasa vasorum occurs inward, and the inflammatory lesion expands along the elastic lamina.

While the arteries that form aneurysms are typically muscular, and occur along with the vasa vasorum (e.g., coronary artery, axillary artery, internal iliac artery), edema and cell infiltration are also seen at arterioles, venules, and microvessels. The pathophysiology of coronary aneurysms remains unclear, however, we hypothesize that both endothelial cell disorders and inflammation of the vasa vasorum are involved. If there is prolonged edema of the microvessels, the supply of oxygen to the coronary artery wall through the vasa vasorum will be compromised, and the coronary media may thus become ischemic. Hence, the coronary artery structure becomes very fragile [37].

As previously noted, we believe that after the development of inflammation around micro arteries in the adventitia, the neovascularization diverges from the vasa vasorum and proliferates toward the media and the intima to supply blood, inflammatory cells, cytokines and chemokines in order to quell the inflammation.

The vasa vasorum have been of considerable interest to scientists and physicians for more than a century [38,39]. They nurture the outer component of the vessel wall, including the adventitia and outer one-third of the media, and the intima is supplied with oxygen from the lumen [30]. Under normal conditions, the vasa vasorum run longitudinally along the long axis of the adventitia of the blood vessels (primary vasa vasorum), and in some places, flow into the adventitia and the media (second vasa vasorum).When any injury or inflammation of blood vessels occurs (e.g., arterial sclerosis), the vasa vasorum proliferate and penetrate into the adventitia [13] and the media, thus buttressing the capillary network [14].

Developments in diagnostic imaging have allowed the microvessels inside lesions to be detected if the neointima is thicker than 500 μm. Furthermore, any infiltration of microvessels from the adventitia into the vessels can now be visualized [15].In inflammatory lesions, the permeability of microvessels can be seen in the acute phase as these microvessels expand or progress. Hypoxia and endothelial reactions are the major driving forces behind the progression of the vasa vasorum [40]. Moreover, there is a disproportionate increase in growth factors for the endothelium of the vasa vasorum, and further development of the vasa vasorum causes the plasma to extravasate around the vasa vasorum.

## Conclusions

In conclusion, we investigated the involvement of the vasa vasorum in the KD like vasculitis using a murine model induced by CAWS. We demonstrated that the vasa vasorum might serve as the initiator of vasculitis in this model. Initially, the inflammatory cells accumulated at the adventitia, and then diverged through neovascularization toward the media and the intima. Therefore, the vasa vasorum provides a critical route for the infiltration of inflammatory cells. The proliferation of the new vasa vasorum toward the media and the intima is an important criterion for both the expansion and the termination of the inflammation.

## Abbreviations

KD: Kawasaki disease; SEM: Scanning electron microscope; CT: Computed tomography; CAWS: *Candida albicans* water-soluble fraction; IVIG: Intravenous immunoglobulin; CAL: Coronary arterial lesion; CADS: *Candida albicans*- derived substance; HE: Hematoxylin and eosin; EVG: Elastica van Gieson.

## Competing interests

The authors declare no conflicts of interests.

## Authors contributions

AHO: Preparation of the murine model and data analysis. CS: Preparation of the murine model and data analysis. TY: Preparation of the murine model and data analysis. KI: Preparation of the murine model and data analysis. NNM: Preparation of the CAWS. NO: Preparation of the CAWS. YA: Analysis using micro CT. HT: Histological evaluation of vasculitis. TT: Histological evaluation of vasculitis. KH: Preparation of the murine model and data analysis. All authors read and approved the final manuscript.

## References

[B1] Kawasaki T Acute febrile mucocutaneous syndrome with lymphoid involvement with specific desquamation of the fingers and toes in children Jpn J Allergol 1967 16 178 222 [in Japanese] 6062087

[B2] Fujiwara H Hamashima Y Pathology of the heart in Kawasaki disease Pediatrics 1978 61 100 107 263836

[B3] Naoe S Shibuya K Takahashi K Wakayama M Masuda H Tanaka N Pathological observation concerning the cardiovascular lesions in Kawasaki disease Cardiol Young 1991 1 206 212

[B4] Yanagawa H Nakamura Y Yashiro M Results of the nationwide epidemiologic survey of Kawasaki disease in 1999 and 2000 in Japan J Pediatr Pract 2002 65 332 342 [in Japanese]

[B5] Akagi T Rose V Benson LN Newman A Freedome RM Outcome of coronary artery aneurysms after Kawasaki disease J Pediatr 1992 121 689 694 10.1016/S0022-3476(05)81894-3 1432415

[B6] Kato H Sugimura T Akagi T Sato N Hashino K Maeno Y Kazue T Eto G Yamaoka R Long-term consequences of Kawasaki disease: a 10- to 21-year follow-up study of 594 patients Circulation 1996 94 1379 1385 10.1161/01.CIR.94.6.1379 8822996

[B7] Onouchi Z Hamaoka K Sakata K Ozawa S Shiraishi I Itoi T Kiyosawa N Long-term changes in coronary artery aneurysms in patients with Kawasaki disease; comparison of therapeutic regimens Circ J 2005 69 265 272 10.1253/circj.69.265 15731529

[B8] Tanaka K Onouchi Z Tomisawa M Goto M Kimpara K Kusunoki T Fukuda M Takeoka S Fujita T The study in MLNS (II). An autopsy case and reference with infantile polyarteritis nodosa Acta Paediat Jap 1973 77 397 411 [in Japanese]

[B9] Onouchi Z Tomizawa M Goto M Nakata K Fukuda M Goto M Cardiac Involvement and Prognosis in Acute Mucocutaneous Lymph Node Syndrome Chest 1975 68 297 301 10.1378/chest.68.3.297 1157533

[B10] Takahashi K Oharaseki T Wakayama M Yokouchi Y Naoe S Murata H Histopathological features of murine systemic vasculitis caused by Candida albicans extract - an animal model of Kawasaki Disease Inflamm Res 2004 53 72 77 10.1007/s00011-003-1225-1 15021972

[B11] Ishida-Okawara A Nagi-Miura N Oharaseki T Takahashi K Okumura A Tachikawa H Kashiwamura S Okamura H Ohno N Okada H Ward PA Suzuki K Neutrophil activation and arteritis induced by C. albicans water-soluble mannoprotein-β-glucan complex (CAWS) Exp Mol Pathol 2007 82 220 226 10.1016/j.yexmp.2006.05.006 17208225 PMC7126757

[B12] Duong TT Silman ED Bissessar MV Yeung RS Superantigenic activity is responsible for induction of coronary arteritis in mice; an animal model of Kawasaki disease Int Immunol 2003 15 79 89 10.1093/intimm/dxg007 12502728

[B13] Barger AC Beeuwkes R 3rd Lainey LL Silverman KJ Hypothesis: Vaso vasorum and neovascularization of human coronary arteries. A possible role in the pathophysiology of atherosclerosis N Eng J Med 1984 310 175 177 10.1056/NEJM198401193100307 6197652

[B14] Moulton KS Plaque angiogenesis: Its functions and regulation Cold Spring Harb Symp Quant Biol 2002 67 471 482 10.1101/sqb.2002.67.471 12858573

[B15] Zhang Y Cliff WJ Schoefl GI Higgins G Immunohistochemical study of intimal microvessels in coronary atherosclerosis Am J Pathol 1993 143 164 172 7686341 PMC1886935

[B16] Murata H Experimental candida-induced arteritis in mice. Relation to arteritis in the mucocutaneous lymph node syndrome Microbiol Immunol 1979 23 825 831 10.1111/j.1348-0421.1979.tb02815.x 395420

[B17] Murata H Iijima H Naoe S Atobe T Uchiyama S Arakawa S The pathogenesis of experimental arteritis induced by Candida alkali extract in mice Jpn J Exp Med 1987 57 305 313 3330145

[B18] Kurihara K Miura NN Uchiyama M Ohno N Adachi Y Aizawa M Tamura H Tanaka S Yadomae T Measurement of blood clearance time by Limulus G test of Candida water-soluble polysaccharide fraction, CAWS, in mice FEMS Immunol Med Microbiol 2000 29 69 76 10.1111/j.1574-695X.2000.tb01507.x 10967263

[B19] Kurihara K Shingo Y Miura NN Horie S Usui Y Adachi Y Yadomae T Ohno N Effect of CAWS, a mannoseprotein-beta-glucan complex of Candida albicans, on leukocyte, endothelial cell, and platelet functions in vitro Bil Pharm Bull 2003 26 233 240 10.1248/bpb.26.233 12576686

[B20] Uchiyama M Ohno N Miura NN Adachi Y Aizawa MW Tamura H Tanaka S Yadomae T Chemical and immunochemical characterization of limulus factor G-activating substance of Candida spp FEMS Immunol Med Microbiol 1999 24 411 420 10.1111/j.1574-695X.1999.tb01313.x 10435760

[B21] Ohno N Chemistry and biology of angitis inducer, Candida albicans water-soluble mannoprotein-beta-glucan complex (CAWS) Microbiolo Immunol 2003 47 479 490 10.1111/j.1348-0421.2003.tb03409.x 12953841

[B22] Nagi-Miura N Shingo Y Adachi Y Ishida-Okawara A Oharaseki T Takahashi K Naoe S Suzuki K Ohno N Induction of coronary arteritis with administration of CAWS (Candida albicans water-soluble fraction) depending on mouse strains Immunopharmacol Immunotoxicol 2004 26 527 543 10.1081/IPH-200042295 15658603

[B23] Ohno N Murine Model of Kawasaki Disease Induced by Mannoprotein-β-Glucan Complex, CAWS, Obtained from Candida albicans Jpn J Infect Dis 2004 57 S9 S10 15507772

[B24] Nagi-Miura N Harada T Shinohaara H Kurihara K Adachi Y Ishida-Okawara A Oharaseki T Takahashi K Naoe S Suzuki K Ohno N Lethal and severe coronary arteritis in DBA/2 mice induced by fungal pathogen, CAWS, Candida albicans water-soluble fraction Atherosclerosis 2006 186 310 320 10.1016/j.atherosclerosis.2005.08.014 16157343

[B25] Hirata N Ishibashi K Ohta S Hata S Shinohara H Kitamura M Miura N Ohno N Histopathologcal Examination and Analysis of Mortality in DBA/2 Mouse Vasculitis Induced with Caws, a water-soluble Extracellular Polysaccharide Fraction Obtained from Candida albicans Yakugaku Zasshi 2006 126 643 650 [in Japanese] 10.1248/yakushi.126.643 16880722

[B26] Tanaka K Nagata D Hirata Y Tabata Y Nagai R Sata M Augmented angiogenesis in adventitia promotes growth of atherosclerotic plaque in apolipoprotein E-deficient mice Atherosclerosis 2011 215 366 373 10.1016/j.atherosclerosis.2011.01.016 21306712

[B27] Arai Y Yamada A Ninoyama T Kato T Masuda Y Micro-computed tomography newly developed for in vivo small animal imaging Oral Radiology 2005 21 14 18 10.1007/s11282-005-0024-5

[B28] Kwon HM Sangiorgi G Ritman EL McKenna C Holmes DR Jr Schwartz RS Lerman A Enhanced coronary vasa vasorum neovascularization in experimental hypercholesterolemia J Clin Invest 1998 101 1551 1556 10.1172/JCI1568 9541483 PMC508734

[B29] Herrmann J Lerman LO Rodriguez-Porcel M Holmes DR Jr Richardson DM Ritman EL Lerman A Coronary vasa vasorum neovascularization precedes epicardial endothelial dysfunction in experimental hypercholesterolemia Cardiovasc Res 2001 51 762 766 10.1016/S0008-6363(01)00347-9 11530109

[B30] Heistad DD Marcus ML Law EG Armstrong ML Ehrhardt JC Abboud FM Regulation of blood flow to the aortic media in dogs J Clin Invest 1978 62 133 140 10.1172/JCI109097 659626 PMC371746

[B31] Heistad DD Marcus ML Larsen GE Armstrong ML Role of vasa vasorum in nourishment of the aortic wall Am J Physiol 1981 240 H781 H787 7235037 10.1152/ajpheart.1981.240.5.H781

[B32] McGeachie J Campbell P Simpson S Prendergast F Arterial vasa vasorum: a quantitative study in the rat J Anat 1982 134 193 197 7076548 PMC1167909

[B33] Bayer IM Caniggia I Adamson SL Langille BL Experimental angiogenesis of arterial vasa vasorum Cell Tissue Res 2002 307 303 313 10.1007/s00441-002-0512-4 11904766

[B34] Langheinrich AC Kampschulte M Buch T Bohle RM Vasa vasorum and atherosclerosis- Quid novi? Thromb Haemost 2007 97 873 879 17549287

[B35] Moulton KS Vakili K Zurakowski D Soliman M Butterfield C Sylvin E Lo KM Gillies S Javaherian K Folkman J Inhibition of plaque neovascularization reduces macrophage accumulation and progression of advanced atherosclerosis Proc Natl Acad Sci 2003 100 4736 4741 10.1073/pnas.0730843100 12682294 PMC153625

[B36] Endemann DH Schiffrin EL Endothelial dysfunction J Am Soc Nephrol 2004 15 1983 1992 10.1097/01.ASN.0000132474.50966.DA 15284284

[B37] Terai M Pathogenesis of vascular lesions in Kawasaki disease: Clinic and Basic Annual Review JUNKANKI 2004 68 72

[B38] Ritman EL Lerman A The Dynamic Vasa Vasorum Cardiovasc Res 2007 75 4 649 658 10.1016/j.cardiores.2007.06.020 17631284 PMC2121590

[B39] Mulligan- Kehoe MJ The vasa vasorum in diseased and nondiseased arteries Am J Physiol Heart Circ Physiol 2010 298 H295 H305 10.1152/ajpheart.00884.2009 19940078 PMC2822580

[B40] Moreno PR Purushothaman KR Zias E Sanz J Fuster V Neovascularization in human atherosclerosis Circulation 2006 113 2245 2252 10.1161/CIRCULATIONAHA.105.578955 16684874

